# Chronically reduced IL-10 plasma levels are associated with hippocampal sclerosis in temporal lobe epilepsy patients

**DOI:** 10.1186/s12883-020-01825-x

**Published:** 2020-06-12

**Authors:** Pabitra Basnyat, Marko Pesu, Mikael Söderqvist, Anna Grönholm, Suvi Liimatainen, Maria Peltola, Jani Raitanen, Jukka Peltola

**Affiliations:** 1 Department of Neurology, Faculty of Medicine and Health Technology, Tampere University, Arvo Ylpön katu 34, D532, 33520 Tampere, Finland; 2 Department of Neurology, Tampere University Hospital, Tampere, Finland; 3 Immunoregulation, Faculty of Medicine and Health Technology, Tampere University, Tampere, Finland; 4 Fimlab Laboratories, Tampere, Finland; 5 Department of Child Psychiatry, Tampere University Hospital, Tampere, Finland; 6 Faculty of Social Sciences, Health Sciences, Tampere University, Tampere, Finland; 7 UKK Institute for Health Promotion Research, Tampere, Finland

**Keywords:** Epilepsy, Hippocampal sclerosis, Interleukin 10 (IL-10), Refractory, Seizures, Temporal lobe

## Abstract

**Background:**

Increasing evidence supports the role of soluble inflammatory mediators in the pathogenesis of refractory temporal lobe epilepsy (TLE). Hippocampal sclerosis (HS) is a well-described pathohistological abnormality in TLE. The association of proinflammatory cytokines with epileptic disease profiles is well established; however, the potential significance of circulating interleukin 10 (IL-10), particularly in TLE-associated HS, is still poorly understood. Therefore, taking into consideration the neuroprotective and anticonvulsive effects of IL-10, we performed this study to examine the role of the plasma levels of IL-10 in patients with TLE with HS (TLE + HS), TLE without HS (TLE-HS) and with other types of epilepsy.

**Methods:**

This study included 270 patients with refractory epilepsy who were classified into four groups: i) 34 patients with TLE + HS, ii) 105 patients with TLE-HS, iii) 95 patients with extra-TLE (XLE) and iv) 36 patients with idiopathic generalized epilepsy (IGE). The plasma IL-10 levels were quantified using a commercially available enzyme-linked immunosorbent assay (ELISA).

**Results:**

IL-10 levels were significantly lower in TLE + HS than in TLE-HS (*p* = 0.013). In a subgroup of TLE-HS patients who had seizures 1 month before sampling, patients with seizures had significantly higher IL-10 levels than patients who were seizure-free (*p* = 0.039). Among a small group (*n* = 15) of non-refractory TLE-HS patients, IL-10 levels showed a moderate negative correlation with the duration of epilepsy (*r* = − 0.585, *p* = 0.023).

**Conclusions:**

This study demonstrated that chronically reduced levels of plasma IL-10 were associated with HS in TLE patients, suggesting that there was an inadequate systemic anti-inflammatory immune response. These results could provide new biological insights into the pathophysiology of HS in TLE. We also found that the production of IL-10 could be affected by the seizure frequency and declined concomitantly with increased disease durations. Therefore, the measurement of plasma IL-10 may have diagnostic value as a biomarker for stratifying TLE + HS from other epilepsy types or as a marker of disease progression towards a progressive form of epilepsy.

## Background

Epilepsy is a neurological disorder characterized by a chronic predisposition for recurrent unprovoked seizures [1]. Growing evidence suggests that inflammatory mediators play an important role in the underlying pathophysiology of epilepsy, contributing to the onset and perpetuation of seizures [2]. Chronic inflammatory characteristics, such as the infiltration of leukocytes, reactive gliosis, and the overexpression of cytokines, were reported in surgically resected brain tissue from patients with refractory focal epilepsy, supporting a link between inflammation and epilepsy [3].

Cytokines are signalling molecules that are involved in the regulation of immune activation and cell growth, differentiation or death. Among the variety of cytokine classes, interleukins (ILs) are widely studied in epilepsy. The level of these molecules are altered during inflammation, are associated with seizure susceptibility and are possibly involved in epileptogenesis [4]. Based on our previous findings, patients with refractory temporal lobe epilepsy (TLE) had chronically increased concentrations of the proinflammatory cytokine IL-6 in their serum compared with those in healthy controls [5]; these patients also had a postictal increase in plasma IL-6 [6]. Recently, we also showed that IL-6 is increased in the plasma of TLE patients compared to extra-temporal lobe epilepsy (XLE) patients, suggesting that the epilepsy type is a major factor in the seizure-induced production of IL-6 [7]. The putative role of other cytokines, such as TNF-α, IL-1β, and IL-1 receptor antagonist (IL-1RA), in the immunological activation of different stages of seizure disorders was also studied [8].

IL-10 is expressed by a wide variety of immune cells in both the adaptive (Th1, Th2 and Th17 cell subsets, regulatory T cells, CD8+ T cells and B cells) as well as the innate immune system (dendritic cells, macrophages, mast cells, natural killer cells, eosinophils and neutrophils) [9, 10]. Consistent with its role in the periphery, in the CNS, IL-10 inhibits the production of proinflammatory cytokines by microglia, induces the production of transforming growth factor-β by astrocytes, promotes neuronal cell survival and regulates adult neurogenesis [9].

Several studies have demonstrated that IL-10 is associated with a variety of neurological diseases, such as multiple sclerosis (MS), Alzheimer’s disease, Parkinson’s disease and systemic lupus erythaematosus [9]. However, clinical studies describing the association of the anti-inflammatory cytokine IL-10 with different refractory focal epilepsies are still limited. Therefore, taking into consideration the potential neuroprotective and anticonvulsive effects of IL-10 [4], we performed this study to examine the role of circulating IL-10 in patients with refractory TLE with hippocampal sclerosis (TLE + HS), TLE without HS (TLE-HS) and other epilepsies.

## Methods

### Patients

This study included 270 epileptic patients who were classified into four groups based on the anatomical origin of the disease: i) 34 patients with TLE + HS, ii) 105 patients with TLE-HS, iii) 95 patients with XLE and iv) 36 patients with idiopathic generalized epilepsy (IGE). The XLE group consisted of 61 patients with frontal lobe epilepsy (FLE), 9 with parietal lobe epilepsy (PLE), and 7 with occipital lobe epilepsy (OLE). In addition, 7 patients with multifocal epilepsy and 11 patients with focal epilepsies with unknown lobes of onset were categorized as XLE. The idiopathic generalized epilepsy (IGE) group consisted of one patient with juvenile absence epilepsy (JAE), 25 with juvenile myoclonic epilepsy (JME) and 10 with undefined IGE. The classification of epilepsy types was re-evaluated for the purpose of this study. The clinical characteristics of the patients are summarized in Table 1. Epilepsy was defined as refractory epilepsy if seizures persisted after trials with at least two antiepileptic drugs (AEDs) at the maximally tolerated doses, sequentially or in combination [11]. The aetiology was defined according to the findings of magnetic resonance imaging (MRI), the histological analysis of resected lesions and the medical history. Evaluation of available clinical data on the patients revealed that 23 patients had concomitant autoimmune disorders (11 Celiac disease (CD), 1 CD + Sjogren’s syndrome, 4 Multiple sclerosis, 2 Thyroiditis, 1 Type 1 Diabetes mellitus, 1 Gilbert’s syndrome, 1 Lymphocytic colitis + Immunoglobulin A deficiency, 1 Psoriasis and 1 Rheumatoid arthritis). All patients gave written informed consent before blood sampling. This study was conducted at the Outpatient Department of Neurology and Rehabilitation of Tampere University Hospital, Tampere, Finland. This study was approved by the Ethics Committee of Tampere University Hospital.

**Table 1:** Clinical characteristics of patients

	TLE + HS	TLE-HS	XLE	IGE
n	34	105	95	36
Female	21 (61.8)	48 (45.7)	49 (51.6)	22 (61.1)
Age, years	45.1 (13.4)	40.6 (13.9)	37.0 (14.2)	30.7 (12.1)
Age at onset, years	12.9 (9.5)	18.7 (13.1)	16.2 (15.5)	13.9 (7.0)
**Known remote symptomatic aetiology**				
Known aetiology	34 (100)	60 (57.1)	57 (60.0)	0
Genetic	0	0	0	36 (100)
Duration of epilepsy, years	32.1 (14.9)	22.4 (16.9)	20.8 (14.3)	16.6 (11.8)
Patients who fulfilled the criteria for refractory epilepsy	32 (94.1)	89 (84.8)	83 (87.4)	28 (77.8)
Seizure-free patients for 1 year before labs	3 (8.8)	17 (16.1)	15 (15.7)	15 (41.6)
At least one seizure 1 month before labs	25 (73.5)	69 (65.7)	60 (63.2)	14 (38.9)
Seizure frequency 1 month before labs	2.9 (2.9)	4.7 (10.0)	7.7 (15.0)	0.8 (1.4)
Autoimmune disease	5 (14.7)	10 (9.5)	6 (6.3)	2 (5.6)
**Surgery**				
No surgery	26 (76.5)	84 (80.0)	82 (86.3)	36
Epilepsy surgery	8 (23.5)	11 (10.5)	2 (2.1)	0
Other lesional surgery	0	10 (9.5)	11 (11.5)	0

Presented as the mean and standard deviation (for age, age at onset and the duration of epilepsy) or the frequency and proportion (for the rest of the variables)

*TLE + HS* Temporal lobe epilepsy with hippocampal sclerosis, *TLE-HS* Temporal lobe epilepsy without hippocampal sclerosis, *XLE* Extra-temporal lobe epilepsy, *IGE* Idiopathic generalized epilepsy

### Plasma IL-10 quantification

Blood samples were collected during scheduled outpatient visits. Blood was collected in a vacutainer EDTA vacuum tube, centrifuged at 3000 rpm for 10 min, and the separated plasma samples were frozen and stored at − 70 °C until use. IL-10 concentrations in the plasma were measured using commercially available enzyme-linked immunosorbent assay (ELISA) kits according to the manufacturer’s protocol (Pelikine Compact, Sanquin, Amsterdam, The Netherlands).

### Statistical analysis

The clinical characteristics of the patients are reported as the means and standard deviations (SDs) or the frequencies and proportions. Continuous variable data were analysed using the nonparametric Kruskal-Wallis test or Mann–Whitney U test. Spearman’s correlation coefficient was used to analyse the correlation between the IL-10 levels and clinical characteristics of patients. All statistical analyses were performed using Stata statistical software version 13.1 (StataCorp, College Station, Texas, USA). Receiver operating characteristic (ROC) curve analysis was used for evaluating the diagnostic power of IL-10. A *p*-value ≤0.05 was considered significant. Figures were prepared using GraphPad Prism 5.02 software (GraphPad Software Inc., La Jolla, CA, USA).

## Results

### Patients

TLE + HS patients were older and had a longer epilepsy duration than patients with other epilepsies (*p* < 0.001). The age at disease onset was younger for the TLE + HS group than for the TLE-HS group (*p* = 0.040), but the age at disease onset was not different in the TLE + HS, XLE and IGE groups (*p* > 0.05). The total number of seizures during the past 12 months before the lab analyses and a month before the lab analyses was lower for the IGE group than for all other study groups (*p* = 0.003). However, the total number of seizures was similar among TLE + HS, TLE-HS and XLE patients.

### Assessment of IL-10 levels in different patient cohorts

**Fig. 1:**
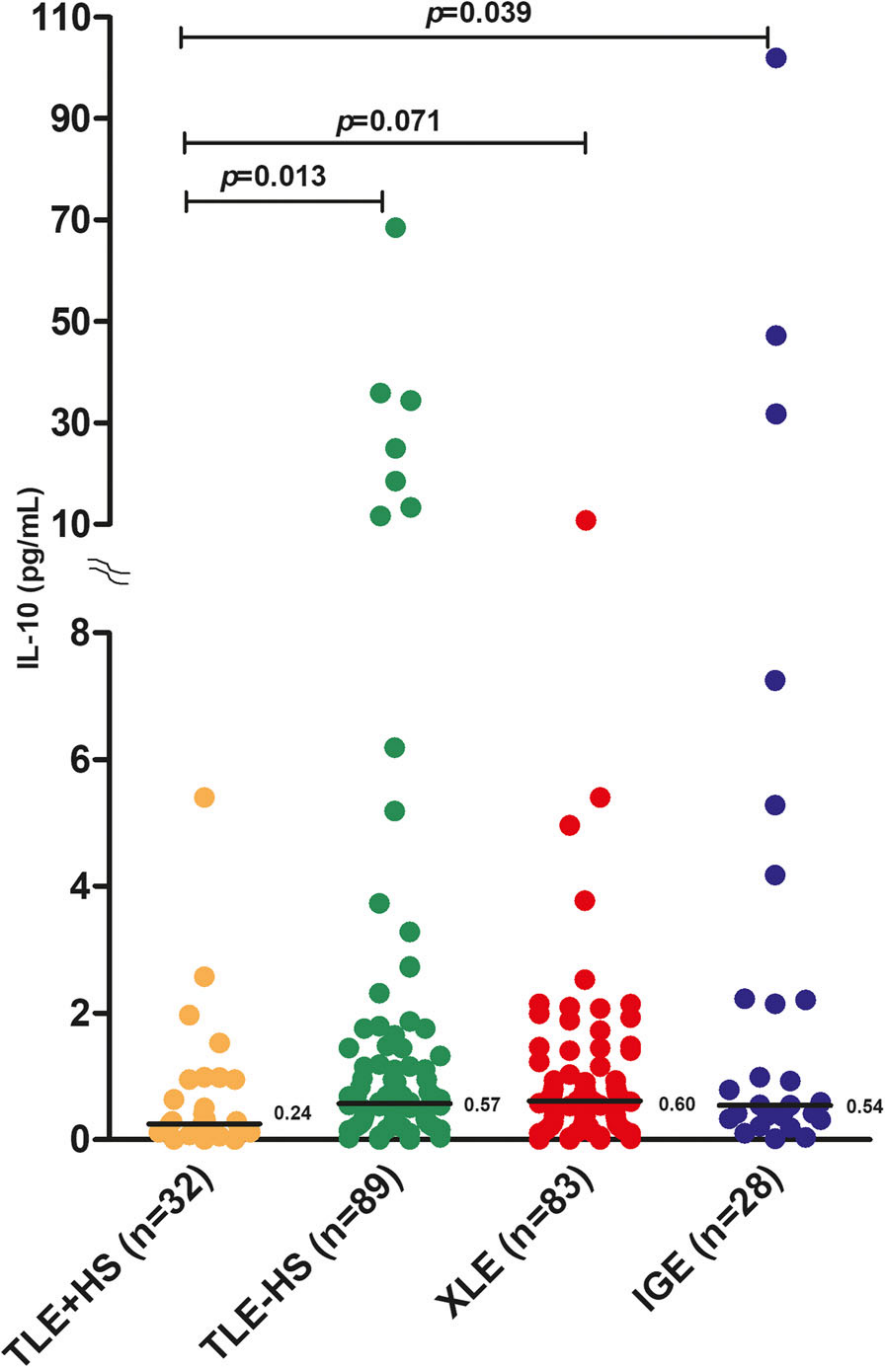
Median IL-10 levels among refractory patients in each epilepsy type. TLE + HS: temporal lobe epilepsy with hippocampal sclerosis; TLE-HS: TLE without HS; XLE: extra-temporal lobe epilepsy; IGE: idiopathic generalized epilepsy

Among the study groups, the plasma levels of IL-10 were significantly lower in the TLE + HS group than in the TLE-HS group (*p* = 0.035) but were not lower than those in the XLE group (*p* = 0.126). The difference in IL-10 levels between TLE + HS and IGE groups showed a trend towards statistical significance (*p* = 0.066). In the current study, 32 (94.1%) TLE + HS patients, 89 (85.6%) TLE-HS patients, 83 (87.3%) XLE patients, and 28 (77.7%) IGE patients were refractory. When we compared IL-10 levels only among drug-resistant patients in each epilepsy types, IL-10 levels were still significantly lower in the TLE + HS group than in the TLE-HS group (*p* = 0.013, Fig. 1) and IGE group (*p* = 0.039). The difference in IL-10 levels between TLE + HS and XLE groups showed a trend towards statistical significance (*p* = 0.071). IL-10 levels were similar in TLE-HS, XLE and IGE groups (*p* > 0.05). There was no significant difference in the IL-10 levels among refractory and non-refractory patients when the whole group was analysed, including in all epilepsy patients, as well as among the individual epilepsy groups (*p* > 0.05). Furthermore, there was no significant difference in the IL-10 levels among the study groups based on the clinical characteristics, such as aetiology (cryptogenic and genetic) or surgery (epileptic surgery, no epileptic surgery and other lesional surgery) (*p* > 0.05). The distribution of IL-10 concentrations among refractory patients in each group is shown in Fig. 1.

**Fig. 2:**
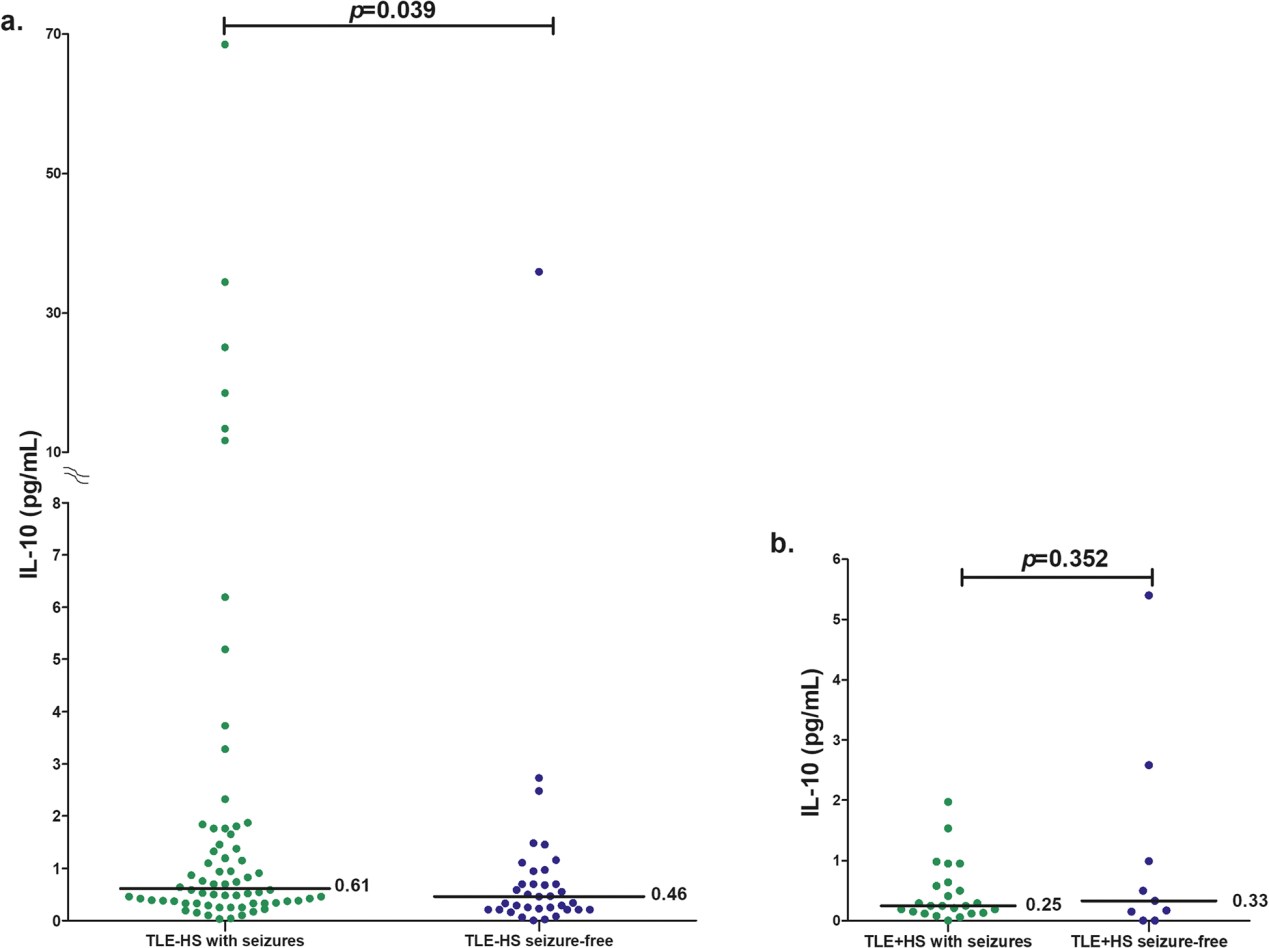
Median IL-10 levels among TLE-HS patients (**a**) and TLE + HS patients (**b**) who had seizures compared to seizure-free patients during the months before sampling. TLE-HS: temporal lobe epilepsy without hippocampal sclerosis; TLE + HS: TLE with HS

In this study, 9 (26.4%) TLE + HS patients, 36 (34.2%) TLE-HS patients, 32 (33.6%) XLE patients, and 21 (60.0%) IGE patients had no seizures for 1 month prior to blood sampling. In subgroup analyses, TLE-HS patients (*n* = 69) who had seizures, median seizure frequency of 4.0 (interquartile range (IQR) 1–5), had significantly higher IL-10 levels than patients who had no seizures for 1 month prior to blood sampling (*p* = 0.039, Fig. 2a). In contrast, the IL-10 levels among TLE + HS patients with seizures tended to be lower than those of patients who had no seizures for 1 month prior to blood sampling; however, the difference was not statistically significant (*p* = 0.352, Fig. 2b). Next, we classified TLE-HS patients who had seizures for 1 month prior to blood sampling into two groups: i) patients with low seizure frequency (≤10 seizures per month, *n* = 58) and ii) patients with high seizure frequency (> 10 seizures per month, *n* = 11). When we compared the IL-10 levels between these two groups, the results showed no difference (*p* = 0.831). Moreover, there were no significant differences in the IL-10 levels among XLE and IGE patients with or without seizures during the month before blood sampling (data not shown).

**Fig. 3:**
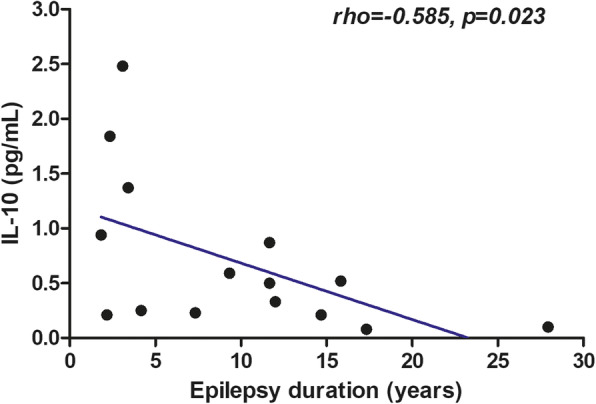
Spearman correlation between IL-10 levels and the duration of epilepsy among non-refractory TLE-HS patients (rho = − 0.585, *p* = 0.023)

Next, we performed correlation analyses to explore the association of IL-10 with several clinical characteristics of epileptic patients in TLE + HS and other study groups. Age, age at disease onset, sex, the frequency of seizures during the month before the labs, the total number of seizures 1 year before the labs and the duration of epilepsy were not correlated with the levels of IL-10. In patients with TLE + HS, IL-10 levels did not differ between males (*n* = 12) and females (*n* = 21) (*p* = 0.274). When analysed, only among refractory patients, as a whole group and groups based on each epilepsy type, IL-10 levels did not correlate with total number of seizures during the month prior to IL-10 analysis (data not shown). Interestingly, among non-refractory TLE-HS patients (*n* = 15), the IL-10 levels showed a moderate negative correlation with the duration of epilepsy (*r* = − 0.585, *p* = 0.023, Fig. 3), and this correlation did not change after adjusting for age (*r* = − 0.536, *p* = 0.042), sex (*r* = − 0.556, *p* = 0.039), and both age and sex (*r* = − 0.549, *p* = 0.052). The mean disease duration of these patients was 9.7 (±7.3 (SD), 1.8–28.0 (range)) years. A similar association did not exist among refractory TLE-HS patients (*r* = − 0.198, *p* = 0.065).

Receiver operating characteristic (ROC) curve analysis was used for each significant comparison in order to understand the value of plasma IL-10 in differentiating the two groups. The results showed only fair diagnostic value of IL-10 in differentiating TLE + HS from TLE-HS (Area under curve (AUC) = 0.692, confidence interval (CI) = 0.57–0.80, *p* = 0.001), XLE (AUC = 0.648, CI = 0.52–0.76, *p* = 0.016), IGE (AUC = 0.701, CI = 0.56–0.83, *p* = 0.004), and also in differentiating TLE-HS patients with seizures from TLE-HS patients who had no seizures for 1 month prior to blood sampling (AUC = 0.624, CI = 0.51–0.73, *p* = 0.039). ROC curves are presented in an additional file (supplementary Fig. 1A-D).

## Discussion

To our knowledge, this is the first clinical study in which plasma IL-10 levels were measured in distinct groups of TLE patients who were stratified based on the presence or absence of HS, including patients with XLE and IGE. In our previous studies, we have shown that there are increased levels of proinflammatory cytokines, particularly IL-6, in TLE [5]; however, in the current study, we detected chronically reduced levels of the anti-inflammatory cytokine IL-10 in TLE patients with HS.

To date, there have been no clinical reports available that we can use as comparisons for our findings in epilepsy. However, a previous study reported IL-10 expression was upregulated in tissue samples from mesial TLE patients with HS (mTLE+HS) compared to tissue samples from mTLE-HS patients and autopsy controls [12]. Other studies have reported similar increases in IL-10 expression in hippocampal tissues from TLE patients compared to tissues from controls [13] and increases in IL-4 in the surgically resected hippocampus of mTLE patients [14]. Although messenger RNA (mRNA) tissue expression and plasma protein levels are not directly comparable, this observation suggests that the systemic production of IL-10 is actually different from the CNS production of IL-10. In other words, the increased IL-10 tissue expression in the brain, as epilepsy-associated alterations, may not be directly reflected in the systemic circulation. TLE is the most common type of focal epilepsy, and HS is a well-described major pathohistological abnormality in refractory TLE that is characterized by the selective loss of neurons [15]. The cause of HS remains elusive; however, multiple factors, such as prolonged febrile seizures, genetic susceptibility, and inflammatory and neurodevelopmental factors, are thought to be involved in HS [16]. Growing evidence supports the role of hippocampal inflammation and blood-brain barrier (BBB) damage in TLE with HS [2]. The upregulation of IL-1β and IL-1 receptor has been demonstrated in astrocytes, microglia and neurons in HS as a result of alterations in BBB permeability [16]. In CNS, microglial cells produce IL-10 upon toll-like receptor (TLR) stimulation, particularly upon TLR2, 3, 4 and 9 stimulation [9]. This TLR-induced production of IL-10 is further regulated by varieties of other molecules present in the microenvironment. In addition to microglia, astrocytes are the other resident CNS cells which produce IL-10 in response to pathogen-associated molecular pattern [17]. Although it is difficult to identify the cellular sources of IL-10 in vivo, several neurodegenerative diseases and animal models of disease have reported the presence of IL-10 in CNS [9]. In epilepsy, specific mechanism by which IL-10 is produced is understudied, probably due to the heterogenous disease pathology. However, in other diseases such as Alzheimer’s disease, IL-10 production occurs through a mechanism involving TLR4 [18]. A recent animal study demonstrated that IL-10 inhibits IL-1β production and inflammasome activation in microglia in epileptic seizures, suggesting the potential of IL-10 in the treatment of epilepsy [19]. Several experimental and clinical studies have demonstrated pharmacological targeting of brain inflammation in epilepsy, for example targeting IL-1R/TLR signaling pathway [20]. This pathway is a key generator of the neuroinflammatory response which is induced in surgically resected epileptogenic foci from patients with refractory epilepsies such as TLE patients with or without HS as compared to control tissue [20]. Of note, in agreement with our findings, progressive MS patients have reported similar decreased levels of circulating IL-10 that are correlated with disease outcomes [21, 22]. A common feature between refractory epilepsy and progressive MS is that the AEDs in epilepsy and anti-inflammatory drugs in MS do not have significant benefit on patients. Progressive MS is a chronic neurodegenerative phase of MS where patients exhibit neurologic deterioration and increasing disability levels [23]. Taken together, reduced IL-10 in TLE + HS patients could be considered a pathophysiological characteristic of the chronic disease state in epilepsy in which an insufficient anti-inflammatory immune response due to neuronal loss and gliosis could be attributed to the reduced IL-10 signalling/bioavailability.

Another noteworthy finding was the increased IL-10 levels among a subgroup of TLE-HS patients who had seizures 1 month prior to the blood sampling compared to those in patients who were seizure-free. These observations indicate that the activation of the immune system during seizure activity leads to the production of the anti-inflammatory cytokine IL-10 at a similar level to proinflammatory cytokines that are produced in an effort to restore immune system homeostasis. In line with this hypothesis, a recent study reported a significant increase in both pro-inflammatory (IFN-γ, IL-6, and IL-8) and anti-inflammatory cytokines (IL-10 and IL-1RA) in the serum of children with febrile seizures [24]. On the other hand, increased IL-10 in patients with seizures may indicate that there is a compensatory activation of the anti-inflammatory pathway to dampen the pro-inflammatory effects. It is important to take into consideration the timing of seizure activity in cytokine analysis because cytokine production varies pre- and post-seizure activity. The effect of seizures on increased IL-10 production 24–72 h after seizure onset was reported in patients with neonatal seizures, indicating the enhanced protective role of IL-10 [25]. A recent report showed that there were unaltered levels of IL-10 among active epilepsy patients with TLE and XLE during the postictal and interictal periods [26]. Therefore, it is equally important to evaluate the effect of seizure activity and the production of IL-10 among a well-characterized TLE cohort during an inpatient video-EEG study, as we have previously performed for IL-6 [6, 7], in future clinical studies. In addition, it is also important to measure IL-10 levels in cerebrospinal fluid (CSF) to evaluate its intrathecal production as we have done previously for IL-6 which showed substantially higher IL-6 in CSF than in plasma [27].

Several cytokines are not present in the circulation during physiological condition or are below the level of detection, however their levels are chronically altered during the pathological conditions. The detection of a negative correlation between IL-10 and the duration of epilepsy may indicate that the production of IL-10 declines concomitantly with the increase in disease duration, regardless of age and gender. Furthermore, a study has reported that the aging does not affect the physiological production of IL-10 in healthy subjects [28]. During our previous analyses with IL-6 in TLE or XLE patients, we could not detect such association with epilepsy duration [5]. However, some studies have displayed a similar negative correlation of the disease duration with IL-10 in the sera of patients with psychiatric disorders [29] and with immune cell infiltrates in the temporal lobe parenchyma of refractory TLE + HS patients [30]. Since the present finding was limited to only 15 non-refractory TLE-HS patients, we were unable to provide a broader explanation for why a similar association was absent in the whole group of epilepsy patients or in patients with only refractory TLE. One distinct feature of these patients was the shorter disease duration compared to that of refractory TLE patients (10 versus 25 years). Similar disease durations have been reported as the average time for focal epilepsy to become refractory [31]. Therefore, this finding may be useful in providing deeper insights into the role of IL-10 in the pathophysiology of refractory TLE. In addition, this correlation was not evident among the TLE + HS group because there were only two patients with non-refractory TLE. While a number of studies have focused on the association of disease profiles with proinflammatory cytokines [4, 8], studies revealing associations with anti-inflammatory cytokines are still limited in epilepsy. Hence, the reciprocal link between IL-10 and the epilepsy duration should be studied further in a prospective follow-up study to determine whether the IL-10 level could be a marker of disease progression towards the progressive stage. Since the correlation is evident only among small patient population, similar analysis should be re-evaluated in a larger cohort including the patients with similar epilepsy type and disease duration. It is still not clear whether epilepsy is a progressive neurodegenerative disease. A recent study showed that the duration of epilepsy was associated with progressive cortical thinning in patients with focal epilepsy, and cortical thinning was accelerated specifically among patients with HS and those who were younger at the onset of seizures [32].

The present study had some limitations. The study lacks IL-10 measurement from healthy controls. Measurement of the physiological concentrations of IL-10 in healthy subjects [28] would have enabled additional analyses on the impact of our current findings in epilepsy patients. We measured only one cytokine at a single time point. Therefore, the inclusion of some proinflammatory cytokines in longitudinal samples in future studies could enable us to monitor and compare the serial changes in plasma cytokine production in terms of the pro- and anti-inflammatory immune responses. Moreover, the clinical data are heterogeneous concerning the aetiology, epilepsy syndrome, seizure frequency, and disease duration, which limits the statistical power when analysing the patient subgroups. These issues should be addressed by examining larger patient cohorts in future studies.

## Conclusions

The results of the present study suggest that chronically reduced levels of the anti-inflammatory cytokine IL-10 were associated with HS in TLE patients, indicating the presence of an inadequate anti-inflammatory immune response. We further found that the production of IL-10 was affected by seizures and observed a negative correlation between IL-10 and the duration of non-refractory TLE. Moreover, we corroborated our previous observation that the extent of the inflammatory immune response differs between types of epilepsies that are associated with different aetiologies. Therefore, the measurement of plasma IL-10 may have diagnostic value as a potential biomarker for stratifying TLE + HS from other epilepsy types or as a marker for disease progression towards the progressive form of epilepsy. Further studies are needed to confirm these preliminary findings including longitudinal follow-up analyses.

## Supplementary information

**Additional file 1.**

## Data Availability

The data in the current study are available on reasonable request from the corresponding author.

## Abbreviations

AEDs: Antiepileptic drugs
BBB: Blood-brain barrier
CNS: Central nervous system
HS: Hippocampal sclerosis
IGE: Idiopathic generalized epilepsy
IL-10: Interleukin-10
TLE: Temporal lobe epilepsy
XLE: Extra-temporal lobe epilepsy
