# Supplementary figures and images for: Chronically reduced IL-10 plasma levels are associated with hippocampal sclerosis in temporal lobe epilepsy patients
Source: BMC Neurol. 2020 Jun 12;20:241. doi: 10.1186/s12883-020-01825-x (PMC7291453; doi:10.1186/s12883-020-01825-x)

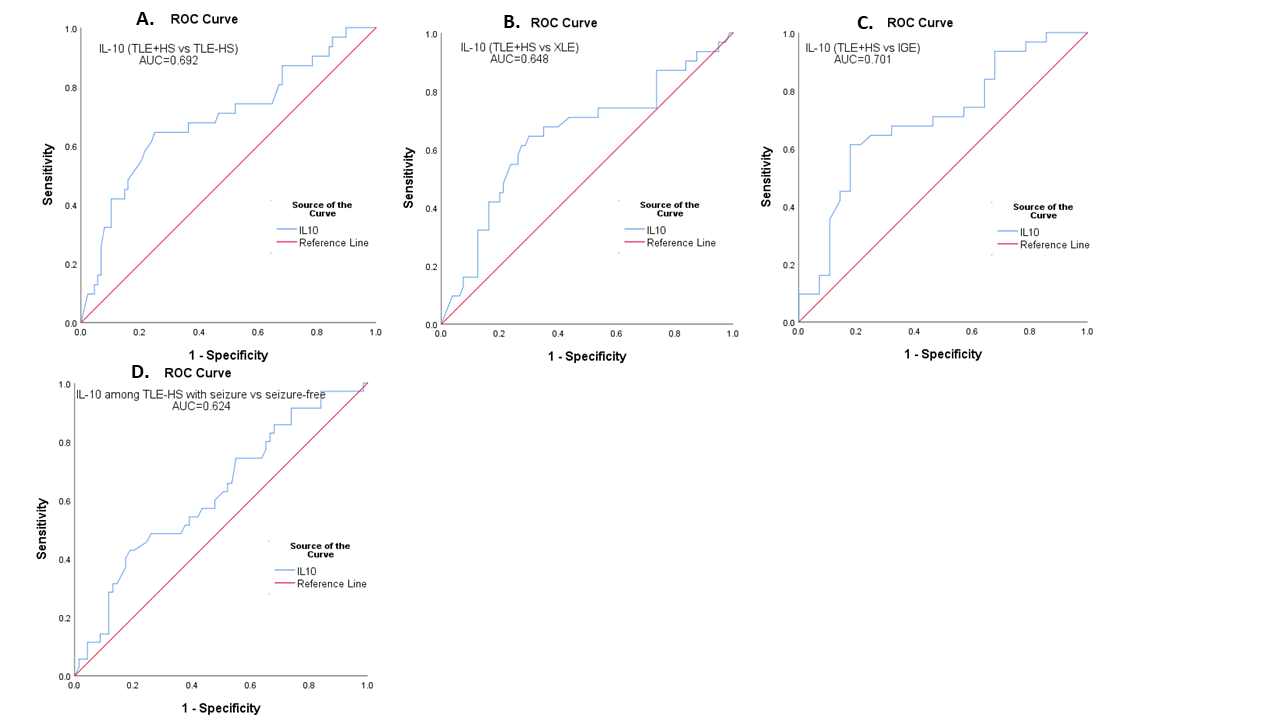

Supplement: Supplementary file 1 — Additional file 1. [file 12883_2020_1825_MOESM1_ESM.tif]
